# Supplementary material for: In vitro culture of bovine fibroblasts using select serum-free media supplemented with Chlorella vulgaris extract
Source: BMC Biotechnol. 2023 Feb 8;23:4. doi: 10.1186/s12896-023-00774-w (PMC9909908; doi:10.1186/s12896-023-00774-w)
Supplement: Supplementary file 1 — Additional file 1. Supplemental data. [file 12896_2023_774_MOESM1_ESM.pdf]

Supplementary Material

In vitro culture of bovine fibroblasts using select serum-free media supplemented with *Chlorella vulgaris* extract

Galileo Defendi-Cho, Timothy M. Gould*

Colorado College, Department of Chemistry and Biochemistry, Colorado Springs, CO 80903.

*Correspondence to: timothymgould@gmail.com

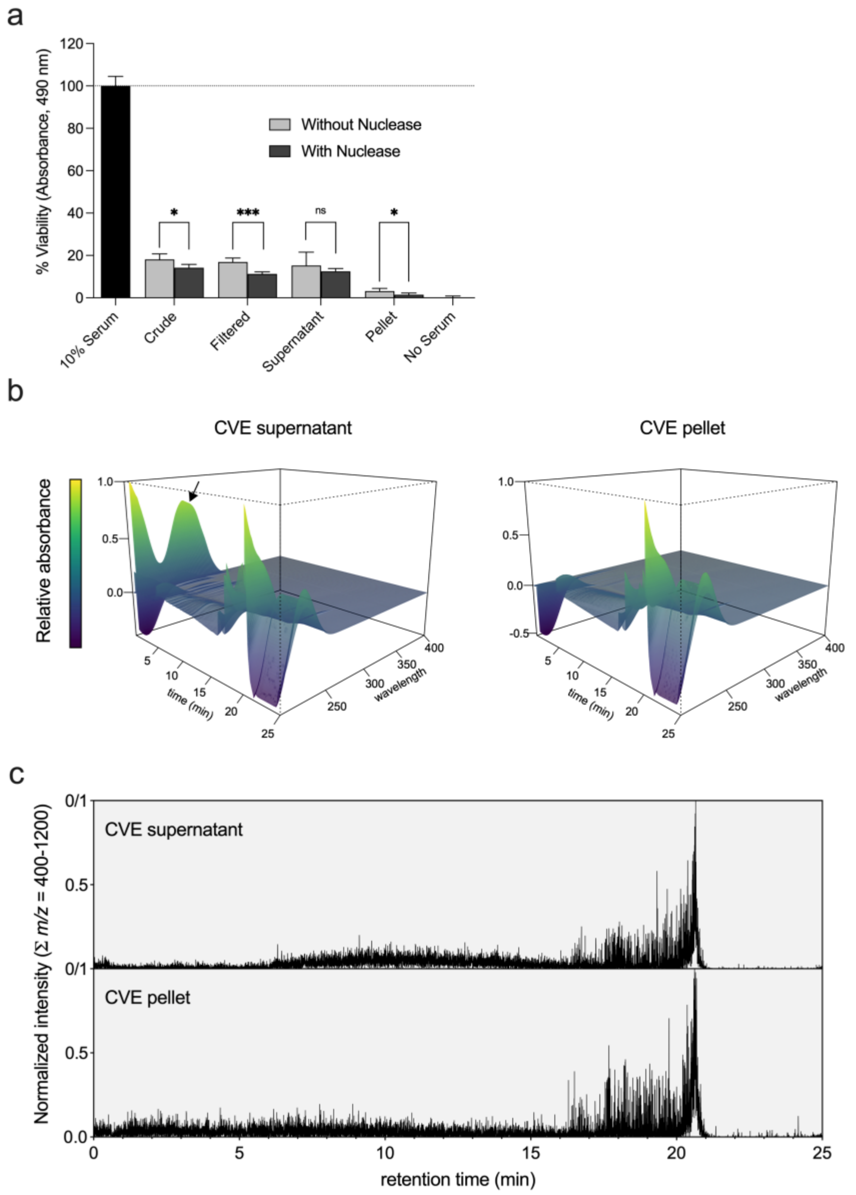

Figure S1: Nuclease-treated *C. vulgaris* extract and its insoluble fraction decrease cell viability. **(a)** Bar plots show relative viability of EBTr cells cultured with different fractions of 10 g/L CVE with and without nuclease treatment relative to control cells cultured in 10% FBS after 3 days. Statistical significance was calculated by t-tests with Welch's correction in which experimental CVE fractionation groups were compared with versus without nuclease treatment; significance levels are indicated as *p<0.05, ***p<0.001, ns=not significant, n=6. **(b)** Perspective 3D surface plots show UV chromatograms of CVE supernatant and pellet fractions (wavelengths 200-400 nm); data shown are mean normalized absorbance spectra from three replicate injections of each sample type. Arrow indicates large, broad peak around 260 nm present in CVE supernatant. Panel **(c)** shows normalized total ion chromatograms (TICs) for the sum of signals of all *m/z* values from 400-1200 for CVE pellet and supernatant; representative data shown are averaged spectra from three injections of the same sample type.

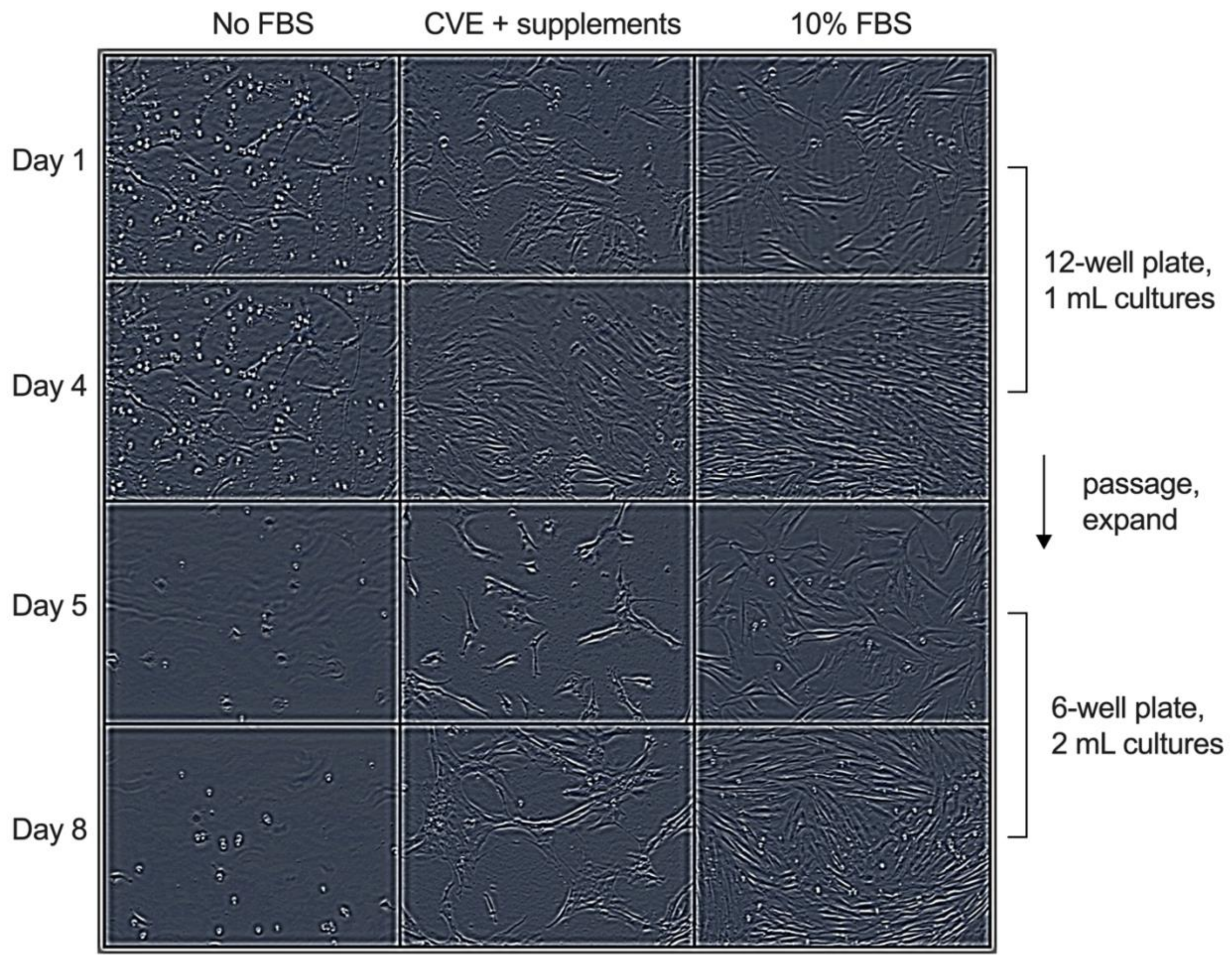

Figure S2: Bovine fibroblasts cultured with select serum-free media and *C. vulgaris* extract show growth and expansion. Brightfield images show representative EBTr cell density and morphology for each condition and time point. CVE was used at 10 g/L; supplements include 10 ng/mL TGF, 30 ng/mL FGF, and 3 μM bovine insulin. Cells were passaged and expanded from 1 to 2 mL cultures (and from 12-well to 6-well plates) immediately after day 4 images were taken.
